# Pathophysiological mechanisms of liver injury in COVID‐19

**DOI:** 10.1111/liv.14730

**Published:** 2020-11-29

**Authors:** Alexander D. Nardo, Mathias Schneeweiss‐Gleixner, May Bakail, Emmanuel D. Dixon, Sigurd F. Lax, Michael Trauner

**Affiliations:** ^1^ Hans Popper Laboratory of Molecular Hepatology Division of Gastroenterology and Hepatology Department of Internal Medicine III Medical University of Vienna Vienna Austria; ^2^ Medical Intensive Care Unit 13H1. Division of Gastroenterology and Hepatology Department of Internal Medicine III Medical University of Vienna Vienna Austria; ^3^ Campus IT Institute of Science and Technology Austria Klosterneuburg Austria; ^4^ Department of Pathology Hospital Graz II Academic Teaching Hospital of the Medical University of Graz Graz Austria; ^5^ School of Medicine Johannes Kepler University Linz Austria

## Abstract

The recent outbreak of coronavirus disease 2019 (COVID‐19), caused by the Severe Acute Respiratory Syndrome Coronavirus‐2 (SARS‐CoV‐2) has resulted in a world‐wide pandemic. Disseminated lung injury with the development of acute respiratory distress syndrome (ARDS) is the main cause of mortality in COVID‐19. Although liver failure does not seem to occur in the absence of pre‐existing liver disease, hepatic involvement in COVID‐19 may correlate with overall disease severity and serve as a prognostic factor for the development of ARDS. The spectrum of liver injury in COVID‐19 may range from direct infection by SARS‐CoV‐2, indirect involvement by systemic inflammation, hypoxic changes, iatrogenic causes such as drugs and ventilation to exacerbation of underlying liver disease. This concise review discusses the potential pathophysiological mechanisms for SARS‐CoV‐2 hepatic tropism as well as acute and possibly long‐term liver injury in COVID‐19.

## 1 INTRODUCTION

Since December 2019, the outbreak of coronavirus disease 2019 (COVID‐19), caused by the novel Severe Acute Respiratory Syndrome (SARS) Coronavirus (CoV) 2 (SARS‐CoV‐2), has led within a few months to a major global health and economic crisis. As of October 2020, more than 40 million confirmed cases have been reported worldwide, with nearly 1 million deaths, affecting 189 countries.^1^ The respiratory tract is considered the main target of SARS‐CoV‐2 infection and a small subset of infected individuals becomes severely ill and may develop acute respiratory distress syndrome (ARDS) with potentially fatal outcome.^2^ More recently, systemic features of the disease with the involvement of organs outside the respiratory tract, including the liver and gastrointestinal tract are receiving increasing attention, indicating that COVID‐19 may be considered as a systemic infectious and inflammatory disease.^3^, ^4^, ^5^, ^6^, ^7^ Although closely related to other Corona virus (CoV) family members SARS‐CoV and MERS‐CoV (Middle East Respiratory Syndrome CoV), infections with the new SARS‐CoV‐2 exhibit a different pathological pattern and the mechanistic link between CoVs‐induced molecular pathophysiological changes and clinical manifestations remains incompletely understood.

**Figure 1:**
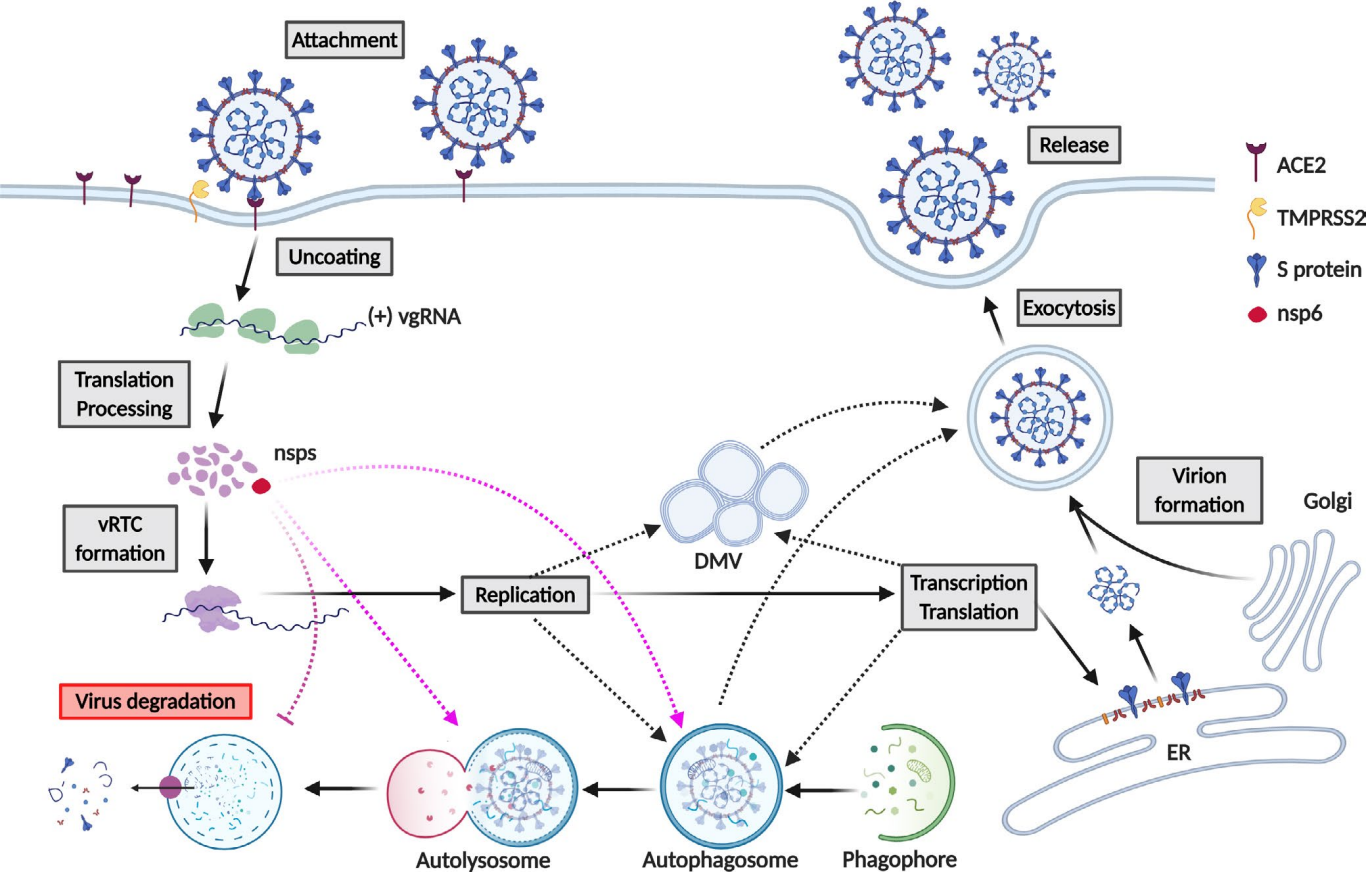
SARS‐CoV‐2 life cycle in host cells. SARS‐CoV‐2 attachment to host cells in liver (eg hepatocytes) may be mediated by the interaction of Spike (S) protein with ACE2. S protein is cleaved by the transmembrane serine protease 2 (TMPRSS2), allowing the cellular entry of the virus. Once uncoated, the viral genome ((+) vgRNA) is released and translated by the ribosome into pp1a and pp1ab (not shown), that are further cleaved into 16 non‐structural proteins (nsps). Following the viral replication/transcription complex (vRTC) assembly, nsp6 (in red) induces autophagosome formation, where viral replication might take place (purple dashed lines). Viral replication might also occur in double‐membrane vesicles (DMV) (black dashed lines). nsp6‐mediated inhibition of autophagosome/lysosome expansion might prevent viral degradation (purple dashed inhibitory line). Newly synthesized viral structural and accessory proteins assemble to form the nucleocapsid and viral envelope at the ER–Golgi intermediate compartment (lower right). Mature virions are then released through the exploitation of the host vesicular system (upper right). DMV and autophagosomes might also be used by the virus for exocytosis and release of mature virions (black dashed lines)

*Coronaviridae* family members, including SARS‐CoV‐2, SARS‐CoV and MERS‐CoV, are enveloped viruses, characterized by a positive single‐stranded RNA genome of about 30Kb.^8^, ^9^, ^10^ The angiotensin‐converting enzyme 2 (ACE2) has been established as the main viral receptor for SARS‐CoV and SARS‐CoV‐2^11^, ^12^ (Figure 1). Following attachment to the host cell and viral S protein priming by the host transmembrane serine protease 2 (TMPRSS2),^13^ SARS‐CoV is internalized by endocytosis and the viral genome is released from the endosome.^14^, ^15^ In the cytosol, the viral RNA is translated into two polyproteins, pp1a and pp1ab, that are further processed to produce 16 non‐structural proteins (nsp1 to nsp16),^16^ the building blocks of the viral replicase–transcriptase complex (RTC).^17^, ^18^ The full viral genome is then replicated in RTC‐containing vesicles.^19^, ^20^ In parallel, a set of specific sub‐genomic mRNA is generated^14^ for the production of SARS‐CoV structural and accessory proteins, which assemble to form the nucleocapsid and viral envelope at the ER–Golgi intermediate compartment, allowing the subsequent release of mature virions^21^ (Figure 1).

Although COVID‐19 primarily affects the respiratory system, emerging evidence highlights the impact of this viral infection on other organ systems.^3^, ^4^, ^5^, ^22^, ^23^ The ubiquitous distribution of the main viral entry receptor ACE2 may explain how SARS‐CoV‐2 is able to cause a widespread disease characterized by systemic organ involvement including the intestines,^24^ heart, kidneys, pancreas, liver, muscular and nervous system.^11^, ^25^, ^26^, ^27^, ^28^ In contrast to SARS‐CoV‐2‐induced lung and myocardial injury, the clinical significance of liver involvement has been controversially debated from the very beginning of the COVID‐19 pandemic.^22^, ^28^, ^29^, ^30^, ^31^, ^32^, ^33^ However, the scientific progress over the last months has shed more light on several key questions concerning COVID‐19‐associated liver injury. In this review, we will highlight molecular evidence pointing towards a putative hepatic tropism of SARS‐CoV‐2, and further review pathophysiological mechanisms that could explain the hepatic phenotypes associated with COVID‐19.

## 2 THE SPECTRUM OF LIVER INVOLVEMENT IN COVID‐19

**Figure 2:**
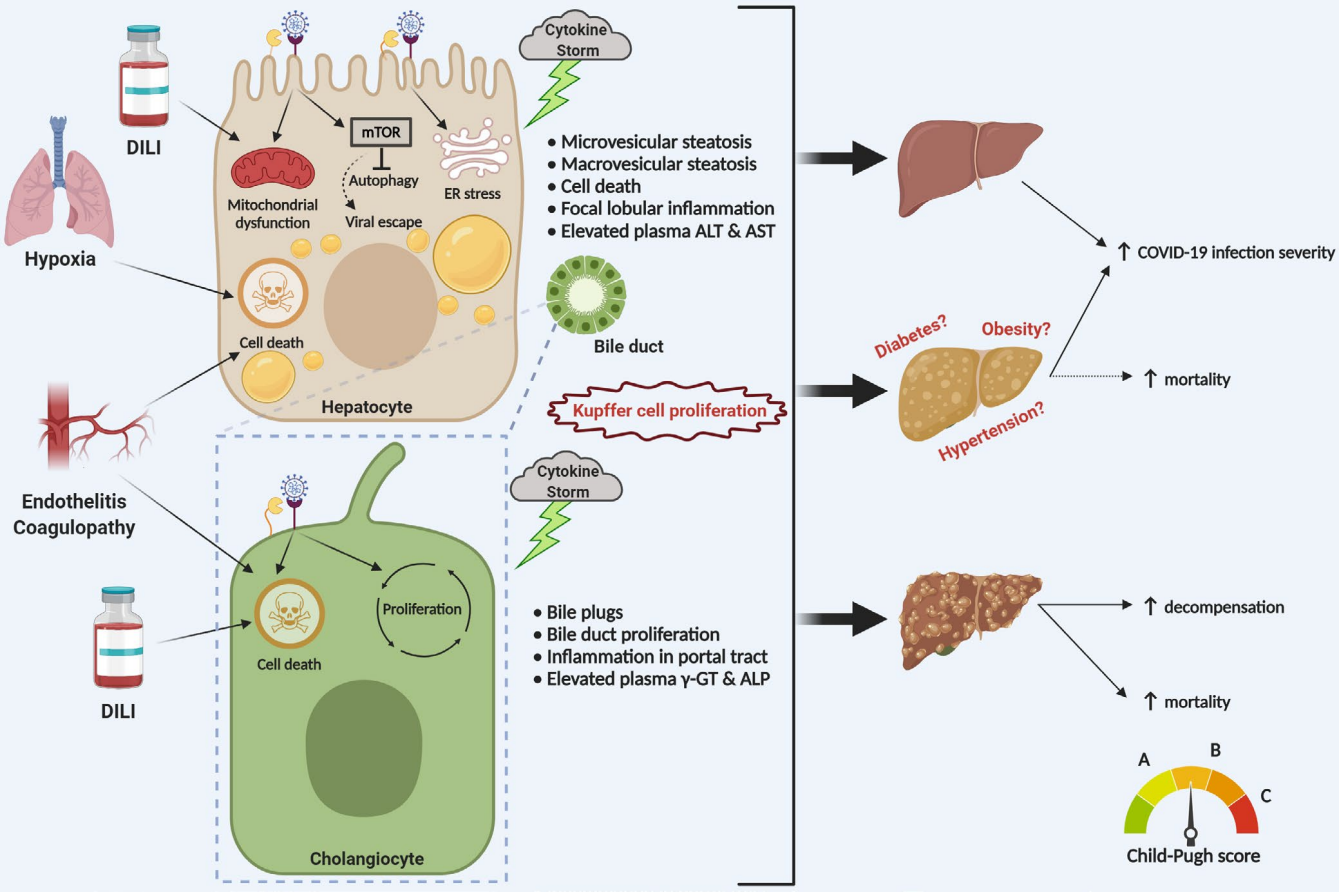
Proposed pathophysiology for liver injury upon SARS‐CoV‐2 infection. COVID‐19‐associated hepatocellular damage is mainly characterized by moderate steatosis, lobular and portal inflammation, apoptotic/necrotic foci and elevation of plasma ALT and AST (upper left panel). Preliminary observations suggest that the injury might be caused by hepatocellular infection with direct cytopathic effects of SARS‐CoV‐2, which could induce mitochondrial dysfunction and ER stress contributing to steatosis. Furthermore, SARS‐CoV‐2 infection might also activate mTOR, which eventually inhibits autophagy (as a mechanism of viral degradation) and facilitates viral escape from the immune system. In addition, cytokine storm, hypoxic conditions due to ARDS and drug‐induced liver injury (DILI) may contribute. COVID‐19‐associated cholangiocellular injury has also been observed and is mainly characterized by bile duct proliferation, occasionally bile plug formation and elevation of plasma γGT and ALP (lower left panel). From a hepatological perspective, COVID‐19‐positive patients may be divided into three categories: patients without pre‐existing chronic liver disease, patients with early stage chronic liver disease and patients with advanced chronic liver disease/cirrhosis. COVID‐19‐associated liver injury may have a more severe outcome in patients with pre‐existing liver disease, such as non‐alcoholic fatty liver disease (NAFLD) and associated metabolic comorbidity. Moreover, COVID‐19 may induce hepatic decompensation with increased mortality in cirrhotic patients (right panel)

COVID‐19 associated liver injury is defined as any liver damage occurring during disease course and treatment of COVID‐19 patients, with or without pre‐existing liver disease.^4^, ^34^, ^35^, ^36^, ^37^, ^38^, ^39^ This includes a broad spectrum of potential pathomechanisms including direct cytotoxicity from active viral replication of SARS‐CoV‐2 in the liver,^40^, ^41^ immune‐mediated liver damage due to the severe inflammatory response/systemic inflammatory response syndrome (SIRS) in COVID‐19,^42^ hypoxic changes induced by respiratory failure, vascular changes due to coagulopathy, endothelitis or cardiac congestion from right heart failure, drug‐induced liver injury and exacerbation of underlying liver disease (Figure 2). The incidence of elevated liver transaminases (ALT and AST) in COVID‐19 patients ranges from 2.5% to 76.3%.^35^, ^38^, ^43^, ^44^ In a recent meta‐analysis, the pooled rate for AST and ALT outside the reference range was 20%‐22.5% and 14.6%‐20.1% respectively.^35^, ^45^ These abnormalities can be accompanied by slightly increased total bilirubin levels in up to 35% of cases.^35^, ^38^, ^43^, ^44^ While elevations of cholestatic liver enzymes [alkaline phosphatase (ALP) and gamma glutamyl transferase (γGT)] were initially considered rather rare,^4^, ^22^, ^23^, ^46^ recent systemic reviews highlight elevations of ALP and γGT in 6.1% and 21.1% of COVID‐19 patients respectively.^35^, ^45^ Moreover, a biphasic pattern with initial transaminase elevations followed by cholestatic liver enzymes has been reported, which could reflect SIRS‐induced cholestasis at the hepatocellular/canalicular level or more severe bile duct injury in the later stage of the disease.^47^ Although COVID‐19‐associated liver injury has been reported to be mild, it may affect a significant proportion of patients, especially those with a more severe disease course. In the light of the central role of the liver for the production of albumin, acute phase reactants and coagulation factors, hepatic dysfunction may impact on the multisystem manifestations of COVID‐19 such as ARDS, coagulopathy and multiorgan failure.^2^, ^3^, ^4^, ^5^, ^6^, ^7^, ^48^ Moreover, the liver is the primary metabolic and detoxifying organ in the human organism, and even a moderate loss of hepatic function could alter the safety profile and therapeutic efficacy of antiviral drugs metabolized in the liver. Hence, it is crucial to understand the causes of COVID‐19‐associated liver injury in more detail.

So far, systematic information on underlying histopathological alterations is scarce. Hepatic steatosis (in part microvesicular) and Kupffer cell activation appear to be commonly encountered in livers of SARS‐CoV‐2‐infected deceased, together with vascular alterations including derangement of intrahepatic portal vein branches, usually mild lobular and portal inflammation, ductular proliferation and liver cell necrosis.^40^, ^46^, ^49^, ^50^, ^51^ Of note, examination of liver biopsies from a cohort of 48 deceased COVID‐19 patients revealed extensive luminal thrombosis at the portal and sinusoidal level, together with portal fibrosis accompanied by significant pericyte activation.^51^

## 3 POTENTIAL MOLECULAR MECHANISMS FOR SARS‐COV‐2 TROPISM OF THE LIVER

The presence of SARS‐CoV‐2 viral RNA has recently been demonstrated by qRT‐PCR in liver among various other organs outside the respiratory tract,^52^ although the exact cellular site of replication remained unspecified since nucleic acids have been isolated by whole‐tissue homogenization. However, in situ hybridization analyses revealed SARS‐CoV‐2 virions in vessel lumens and endothelial cells of portal veins of COVID‐19 liver specimens.^51^ Moreover, electron microscopic analyses on liver samples from two deceased COVID‐19 patients with elevated liver enzymes demonstrated the presence of intact viral particles in the cytoplasm of hepatocytes.^40^

Given recent, although still limited, discoveries,^40^, ^51^, ^52^ hepatic tropism for SARS‐CoV‐2 and direct cytopathic effects should be considered as potential mechanism of COVID‐19 associated liver injury, although a classic hepatitic picture has not been reported.^40^, ^46^, ^49^, ^50^, ^51^ The availability of viral receptors at the host cell surface is a major determinant of viral tropism for a specific tissue.^53^ As such, SARS‐CoV‐2 cell entry is mediated by the S protein of the virus, which specifically interacts with host ACE2 and TMPRSS2 (Figure 1). In order to understand whether SARS‐CoV‐2 might be able to infect liver cells, we explored the expression pattern of the human ACE2 and TMPRSS2 proteins using the Human Protein Atlas (data available at https://www.proteinatlas.org/ENSG00000130234‐ACE2/tissue and https://www.proteinatlas.org/ENSG00000184012‐TMPRSS2/tissue). Interestingly, the expression levels of the two proteins is highest in intestine and gall bladder, but it appears to be virtually absent in the liver. These data might be incomplete or lack sensitivity, since in the Human Protein Atlas ACE2 expression also seems to be absent in the lungs, where infection is definitely known to occur. In a recent study, Chai and colleagues applied single‐cell RNAseq to healthy human liver samples and found that ACE2 expression levels in bile duct epithelium (cholangiocytes) is comparable to that of alveolar cells in the lungs, whereas hepatocellular ACE2 expression is low but still detectable.^54^ Further confirmation of significant ACE2 and TMPRSS2 expression in liver parenchymal cells comes from bio‐informatics analyses from the single‐cell transcriptome database Single Cell Portal.^55^ Interestingly, sinusoidal endothelial cells appear to be ACE2‐negative, in line with previous observations.^56^ This finding may be important considering recent reports on endothelitis of large intrahepatic vessels caused by SARS‐CoV‐2^48^, ^57^ and high ACE2 expression in other endothelia, including central and portal veins, which also can become infected by the virus.^51^

Of note, studies in both mice and humans revealed increased hepatic ACE2 expression in hepatocytes upon liver fibrotic/cirrhotic conditions^58^, ^59^ (and our own unpublished observations). This finding may be of great relevance since pre‐existing liver injury could thereby exacerbate SARS‐CoV‐2 hepatic tropism. Moreover, hypoxia, which is a typical feature in severe COVID‐19 cases, has been shown to be a main regulator of hepatocellular ACE2 expression.^58^ This might explain why extra‐pulmonary SARS‐CoV‐2 dissemination is mainly observed in patients manifesting ARDS and other hypoxic conditions. Importantly, inflammatory conditions/diseases in the liver, as shown for other organs,^60^, ^61^ could also upregulate ACE2 expression. Since drug‐induced liver injury (DILI) may contribute to liver injury in COVID‐19 patients,^62^ it might be of interest to explore whether DILI or certain drugs induce hepatic ACE2 over‐expression.

In vitro experiments also showed that the S protein of lineage B beta‐coronaviruses significantly increases the affinity for its receptor when it is pre‐incubated with trypsin, that is when it is proteolytically activated.^63^ Since liver epithelial cells express trypsin^64^ and a plethora of other serine‐proteases which constantly remodel the extracellular matrix,^65^ ACE2 expression required for SARS‐CoV‐2 target and recognition in the liver might be lower than in other tissues with reduced extracellular proteolytic activity.^66^ In line with these findings, it has been recently discovered that the S protein of SARS‐CoV‐2 bears a furin‐like proteolytic site never observed before in other coronaviruses of the same lineage.^67^ Interestingly, furin is predominantly expressed in organs that have been proposed as permissive for SARS‐CoV‐2 infection, such as salivary glands, kidney, pancreas (data for The Human Protein Atlas, available at https://www.proteinatlas.org/ENSG00000140564‐FURIN/tissue) and the liver.^55^

Finally, other factors, as for example ganglioside (GM1),^68^ might influence S protein‐ACE2 interaction. Therefore, research should also explore more deeply the S protein‐ACE2 interactome to achieve new molecular and therapeutic insights.

In a recent report, Ou and colleagues tested pseudovirions containing the SARS‐CoV‐2 S protein for their ability to infect different cell lines. Interestingly, HuH7 cells, a hepatocyte cell line, as well as Calu3 cells, a human lung carcinoma cell line, were more efficiently transfected by viral vectors carrying the SARS‐CoV‐2 S protein than control pseudovirions.^69^ Moreover, these studies revealed that viral entry might depend on the PIKfyve‐TCP2 endocytotic pathway. A crosscheck in the Human Protein Atlas revealed that both PIKfyve and TPC2 are expressed in liver and gall bladder at comparable levels as in the lung (data available at https://www.proteinatlas.org/ENSG00000115020‐PIKFYVE/tissue and https://www.proteinatlas.org/ENSG00000162341‐TPCN2/tissue), highlighting the potential relevance of this pathway for hepatic tropism, which therefore expands from simple targeting and recognition to support of intracellular viral replication.

In an effort to establish a new and effective functional viromics screening approach aimed at predicting the likelihood of zoonotic events of the known lineage B betacoronaviruses, Letko and colleagues took advantage of HuH7 cells as a permissive model for SARS‐CoV and SARS‐CoV‐2 binding and recognition,^63^ further proving SARS‐CoV‐2 tropism for hepatocytes. Of note, HuH7 cells were described as the third most permissive cell line in this study after pulmonary (Calu3) and intestinal (CaCo2) cell models,^63^ the latter representing organs with histopathologically proven SARS‐CoV‐2 infection. However, the ability of binding and internalizing viral particles does not necessarily imply that the cell type under investigation is also permissive for effective viral replication. In this regard, both Chu and colleagues and Harcourt et al demonstrated that HuH7 cells support SARS‐CoV‐2 viral replication.^70^, ^71^ Hepatocyte cell lines are now such an established permissive cell type for SARS‐CoV and SARS‐CoV‐2 infection that HuH7 cells have also been recently used as positive control in SARS‐CoV‐2 immunostainings.^72^

Although the above‐reported observations define hepatocytes as putative hosts for SARS‐CoV‐2, it is important to point out that all the data arise from studies in which cancer cell lines have been used. In order to clarify the translational potential of these observations, ACE2 protein expression in HuH7 cells should be compared with that of primary human hepatocytes. Furthermore, future investigations are needed to uncover the molecular changes induced in hepatocytes upon SARS‐CoV‐2 infection.

A reliable source of information comes from recent work by Yang and colleagues, who demonstrated SARS‐CoV‐2 tropism for hepatocytes using organoids obtained from human pluripotent stem cell (hPSC)‐derived hepatocyte and primary adult human hepatocytes.^73^ In these systems, pseudovirions expressing SARS‐Cov‐2 S protein were able to infect human hepatocytes, while SARS‐CoV‐2 infection resulted in robust viral replication.^73^ Gene expression analyses also showed that SARS‐CoV‐2‐infected primary hepatocytes over‐express pro‐inflammatory cytokines, while downregulating key metabolic processes, as reflected by the inhibition of CYP7A1, CYP2A6, CYP1A2 and CYP2D6 expression.^73^

Finally, Wang and colleagues applied electron microscopy imaging to liver samples of two deceased COVID‐19 patients, and identified viral structures in hepatocytes which distinctively resemble SARS‐CoV‐2 virions.^40^ This raises the possibility that the histopathological alterations seen in these patients may be caused by direct cytopathic effects of SARS‐CoV‐2^40^although a typical hepatitis pattern appears to be lacking.^40^, ^46^, ^49^, ^50^, ^51^ However, further studies with larger biopsy/autopsy cohorts and the combined imaging (including immune electron microscopy) may be necessary to confirm these preliminary observations of hepatocellular SARS‐CoV‐2 presence.

Bile duct epithelial cells (cholangiocytes) participate in bile production and flow as well in immune response.^74^ Single‐cell sequencing of human long‐term liver ductal organoid cultures showed preservation of ACE2 and TMPRSS2 expression.^75^ Following SARS‐CoV‐2 infection, cholangiocytes underwent syncytia formation and the amount of SARS‐CoV‐2 genomic RNA was dramatically increased 24 hours post‐infection. Similar results have been obtained when infecting adult human cholangiocyte organoids with SARS‐CoV‐2.^73^ These observations indicate that human liver ductal organoids may be susceptible to SARS‐CoV‐2 infection in vitro and suggest that viral replication could also occur within the bile duct epithelium in vivo. However, despite significantly higher ACE2 expression when compared with hepatocytes, no direct evidence of SARS‐CoV‐2 cholangiocellular infection has been reported so far in COVID‐19 patients. Since bile is primarily produced by hepatocytes and cholangiocytes, and given the continuous and direct contact between biliary fluids and the cholangiocellular apical membrane, identification of SARS‐CoV‐2 viral RNA or proteins in bile could be an indirect proof of SARS‐CoV‐2 cholangiocellular infection. At the moment, only one case report has shown SARS‐CoV‐2 RNA in bile,^76^ whereas bile from two other small sample series tested negative.^24^, ^49^ These discrepancies might rely on the fact that the positive‐tested bile sample has been obtained during surgical resolution of bile duct obstruction,^76^ whereas the negatively tested bile was obtained from 48h post‐mortem autopsies.^24^, ^49^

Tight junctions allow cholangiocytes to act as a protective barrier for parenchymal liver cells from toxic bile components. Viral infection with SARS‐CoV‐2 decreased mRNA expression of cholangiocellular tight junction proteins such as claudin 1 *in vitro*,^75^ implicating reduced barrier function of cholangiocytes. This in turn could cause liver injury through leakage of potentially toxic bile into the periductal space and adjacent liver parenchyma. Of note, expression of the bile acid transporters SLC10A2/ASBT and chloride channel ABCC7/CFTR was significantly down‐regulated by SARS‐CoV‐2 infection.^75^ The negative regulation of these hepatobiliary transporters may impair bile acid sensing/signalling by cholangiocytes and bicarbonate secretion, eventually contributing to biliary changes observed in COVID‐19 infection.^49^ Furthermore, cholangiocytes infected with SARS‐CoV‐2 virus upregulated inflammatory pathways, depicting the induction of a reactive cholangiocyte phenotype.^73^ Future studies will have to explore whether and how SARS‐CoV‐2 may alter secretion of pro‐inflammatory and pro‐fibrogenic cytokines and contribute to the ‘reactive cholangiocyte phenotype’, which could propagate inflammation and fibrosis.^74^

Pre‐existing chronic liver diseases seem to be independent risk factors for poor outcome in COVID‐19, and cirrhosis grade has been defined as a predictor of mortality in SARS‐CoV‐2 infected patients^77^ (Figure 2). Activation of hepatic stellate cells plays a paramount role in the progression of chronic liver disease as the main cellular source of fibrosis^78^ and is induced by pro‐inflammatory and pro‐fibrotic cues, such as Angiotensin II, generated by the catalytic action of ACE as part of the pro‐fibrotic branch of the renin‐angiotensin system.^79^ Of note, ACE2 counteracts ACE function by producing the anti‐inflammatory and anti‐fibrotic Angiotensin‐(1‐7) and thereby decreasing the Angiotensin II/Angiotensin‐(1‐7) ratio.^79^ However, ACE2 expression has neither been detected in quiescent, nor in fibrogenic/activated hepatic stellate cells.^58^, ^80^, ^81^, ^82^, ^83^ These findings suggest that these cells may be a rather non‐permissive host for SARS‐CoV‐2. Nevertheless, the pro‐inflammatory milieu generated by direct or indirect COVID‐19‐associated hepatocellular and cholangiocellular injury may pave the way for activation of hepatic stellate cells and consequent induction of fibrosis. This possibility may be even more relevant in patients with underlying CLD, such as NAFLD. Although available data suggest that COVID‐19‐related liver injury is mild and transitory, long‐term follow‐up studies will be necessary to exclude hepatic fibrosis as a potential long‐term consequence of COVID‐19, especially in the presence of pre‐existing liver diseases.

Monocyte‐derived macrophages (MoM) and alveolar macrophages are known to express ACE2,^84^, ^85^ and there is evidence of alveolar macrophage infection by SARS‐CoV^85^ and SARS‐CoV‐2 with detection of viral protein by immunohistochemistry.^24^, ^86^ However, a histopathologic assessment of ACE2 tissue distribution showed no staining in Kupffer cells and other hepatic immune cells,^56^ although Kupffer cell proliferation is typically observed in livers of COVID‐19 diseased.^40^, ^49^ The recent COVID‐19 pandemic further prompted more in‐detail investigations on ACE2 expression and de novo single‐cell RNAseq analyses,^54^ as also in silico evaluations of RNAseq databases^87^, ^88^ proved that Kupffer cells do not express ACE2. It has to be kept in mind, however, that all the described evidences refer to healthy human liver samples. Therefore, quantification of ACE2 expression in samples obtained from patients with underlying chronic liver disease or acute liver injury may be needed to obtain definitive insights into macrophage ACE2 expression patterns.

Of note, upon liver injury and/or Kupffer cell depletion, MoM can invade the liver and efficiently replenish the hepatic resident macrophage population^89^, ^90^, ^91^ (and reviewed in detail in^92^). Although in vitro observations proved that MoM does not support efficient replication of SARS‐CoV (and most probably also SARS‐CoV‐2), infected MoM could act as carriers of the pathogen, favouring infection of the ACE2‐expressing cells in the invaded organ.^93^ Furthermore, Kupffer cell activation and proliferation are frequently observed as a consequence of systemic inflammation and Kupffer cell activation has been reported in the liver specimen of deceased COVID‐19 patients.^40^, ^49^ Thus, although Kupffer cells do not express ACE2, monocytic cells might play a key role in SARS‐CoV‐2‐mediated liver injury by propagation of inflammatory stimuli.

## 4 SARS‐CoV2 AND HEPATIC STEATOSIS

Microvesicular and macrovesicular steatosis have been observed in liver autopsies of COVID‐19 patients who presented with SARS‐CoV‐2 infection as the only risk factor for liver injury, and in some cases, SARS‐CoV‐2 hepatocellular infection has been proven.^40^, ^49^ Importantly, hepatic lipid accumulation as a result of SARS‐CoV‐2 infection must be differentiated from pre‐existing NAFLD, which has been shown to increase the risk for poor outcome in COVID‐19 patients.^50^ Deregulated in host lipid metabolism and mitochondrial activity as a result of potential direct SARS‐CoV‐2 cytopathic effects and/or immunopathology induced by cytokine storm, as well as drug side effects (eg corticosteroids) may be important contributors to the development of hepatic steatosis in COVID‐19 (Figure 2).

Microvesicular steatosis is typically caused by genetic or acquired mitochondrial β‐oxidation defects.^94^ Preliminary observations suggest that SARSR‐CoV‐2 affects mitochondrial activity.^95^ Furthermore, Wang et al also identified mitochondrial crista abnormalities in liver specimen of COVID‐19 patients.^40^ Interestingly, impaired mitochondrial activity has also been implicated in the pathogenesis of NAFLD/NASH.^96^ Thus, SARS‐CoV‐2 infection might even worsen the metabolic state and aggravate pre‐existing NAFLD by these mechanisms.

Endoplasmic reticulum (ER) stress is known to induce de novo lipogenesis in hepatocytes.^97^ Several studies have implicated SARS‐CoV infection in the induction of ER stress. For instance, significant up‐regulation of ER stress markers glucose‐regulated protein 78 (GRP78) and GRP94 has been observed upon SARS‐CoV infection in several cell lines.^98^, ^99^, ^100^ The coronavirus S protein seems to be a major burden for the host ER and might play a key role in ER stress induction.^98^, ^99^ Rearrangement of intracellular membranes by extensive depletion of lipid components from the ER during SARS‐CoV‐2 infection may also contribute to ER stress.^20^ Moreover, the ER stress‐related PERK‐eIF2‐α pathway is over‐activated upon SARS‐CoV infection *in vitro*.^101^ Finally, electron microscopy examinations, which proved SARS‐CoV‐2 hepatocellular infection, reported a pathological ER dilatation in infected hepatocytes,^40^ which most probably will cause ER stress. Collectively, these data could indicate that SARS‐CoV‐2, as other coronaviruses, induces ER stress upon infection, and that the ER stress‐induced de novo lipogenesis could also contribute to the development of steatosis in COVID‐19 patients (Figure 2).

De novo lipogenesis is also induced by the mammalian target of rapamycin (mTOR),^102^ which is also the cardinal regulator of autophagy.^103^ SARS‐CoV has been previously shown to hijack the autophagy pathway through processes that rely on the viral non‐structural protein 6 (nsp6), highly conserved in SARS‐CoV‐2.^104^, ^105^, ^106^ Furthermore, mTOR hyper‐activation has been observed in MERS‐CoV‐infected HuH7 cells, and inhibition of mTOR signalling pathway by rapamycin inhibits viral replication.^107^ Given the recent observations that SARS‐CoV‐2 infection restricts autophagy,^108^ it is tempting to speculate that SARS‐CoV‐2, SARS‐CoV and MERS‐CoV share a similar mTOR‐dependent mechanism of infection. Furthermore, significantly increased mTOR activity has been revealed upon IL‐6 stimulation.^109^ Thus, SARS‐CoV‐2 infection could lead to a hyper‐activation of hepatic mTOR signalling, via direct infection of hepatic cells, or indirect, cytokine storm‐related systemic IL‐6‐dependent effects, which could contribute to the steatotic phenotype in livers of COVID‐19 patients (Figure 2).

Although disadvantageous for the host, induction of host lipogenesis might be crucial for SARS‐CoV‐2 life cycle. Indeed, enhanced de novo lipogenesis could supply the virus with sufficient amounts of lipids to generate the vesicular systems required for viral replication and exocytosis. mTOR‐mediated promotion of protein synthesis^110^, ^111^ and inhibition of autophagolysosome formation^112^, ^113^ may further favour viral replication while preventing viral degradation and ignition of an adequate immune response. Since insulin and glucose signalling positively regulate mTOR activity in the liver,^114^, ^115^ constitutive mTOR over‐activation in obese and diabetic patients^116^, ^117^, ^118^ could at least in part explain their higher risk for worse outcome of COVID‐19 (Figure 2).

## 5 SIRS‐INDUCED CHOLESTASIS AND BILE DUCT ALTERATIONS IN COVID‐19

Cholestatic features such as bile duct proliferation, portal inflammatory infiltrates, and in some cases, canalicular/ductular bile plugs have been reported in post‐mortem evaluations on COVID‐19 patients.^49^, ^119^ The cytokine storm characteristic of the SARS‐CoV‐2‐associated viral sepsis^120^ may be a major contributing factor, since cytokines like TNF‐alpha, IL‐1 and IL‐6 can induce hepatocellular cholestasis by down‐regulating hepatobiliary uptake and excretory systems,^121^, ^122^ resembling the pathomechanisms seen in sepsis‐induced cholestasis.^121^, ^122^, ^123^, ^124^, ^125^ Further studies will have to explore whether—similar to sepsis—serum bile acids as the most accurate indicators of cholestasis may be relevant prognostic parameters in COVID‐19.^122^, ^126^ Sustained systemic IL‐6 signalling initiated by SARS‐CoV‐2 infection induces a C/EBPβ‐dependent suppression of albumin synthesis.^127^ In addition to hypo‐albuminaemia, cholestasis in SIRS as a result of repressed hepatobiliary excretory function could be viewed as part of the negative acute phase response in COVID‐19.

In addition to hepatocellular features, bile duct changes, such as ductular proliferation have been observed in postmortem studies.^49^ Notably, IL‐6 is a strong cholangiocellular mitogen factor^128^ and induces a proliferative and pro‐inflammatory phenotype.^74^, ^129^ Bile ducts from patients with COVID‐19 could therefore be exposed to a ‘triple hit’ from (i) hypoxia from respiratory failure (potentially aggravated by obliteration of the peribiliary arterial plexus through vasculitic/thrombotic changes); (ii) systemic SIRS resulting in a reactive cholangiocyte phenotype or senescence‐associated secretory phenotype, thus actively propagating inflammation as well as fibrosis and (iii) potential viral infection of cholangiocytes themselves. Thus, the hepatobiliary system may become an important target for adverse long‐term hepatic outcomes of COVID‐19. Secondary sclerosing cholangitis of critical ill patients (SSC‐CIP) is a rare but clinically relevant complication in critically ill patients with severe trauma, burn injury, suffering from severe respiratory failure or requiring vasopressor therapy due to hemodynamic instability.^130^, ^131^ Malperfusion and hypoxia, as well as recurrent inflammatory stimuli, are the main triggers for the destruction of the biliary epithelium in SSC‐CIP,^122^ all conditions present in severe COVID‐19 patients.

Therefore, hepatic long‐term follow‐up for COVID‐19 survivors who experienced a severe disease course, such as ARDS with ECMO and prolonged ICU admission might be considered. Early diagnosis is paramount to best manage symptoms and disease progression of SSC‐CIP, which could be counteracted with anti‐cholestatic, cholangio‐protective drugs such as UDCA or more recently norUDCA.^132^, ^133^, ^134^

## 6 SARS‐COV‐2 AND HYPOXIC HEPATITIS

Causes for hypoxic hepatitis are multifactorial. In general, cardiac failure, sepsis and respiratory failure account for more than 90% of all cases.^135^, ^136^, ^137^, ^138^ Additionally, right‐sided heart failure was found to aggravate liver injury by liver congestion as a result of elevated central venous pressure.^122^, ^135^, ^136^, ^137^, ^138^, ^139^, ^140^ In cases of long‐lasting hemodynamic and/or respiratory failure, hypoxia results in hepatic cell death, histopathologically defined as centrilobular necrosis.^141^

COVID‐19‐associated ARDS remains the most common complication requiring critical care management including invasive ventilation, high levels of positive end‐expiratory pressure (PEEP) and vasoconstrictor therapy in case of hemodynamic instability.^142^, ^143^, ^144^, ^145^ These factors may be accompanied by right ventricular dysfunction caused by high pulmonary vascular resistance as a result of hypoxaemia and hypercapnia during ARDS.^146^, ^147^ Furthermore, COVID‐19 causes a hyper‐coagulate state with a significant incidence of pulmonary thrombotic complications aggravating acute right‐sided heart failure and consequently liver congestion.^148^ However, in the majority of cases, SARS‐CoV‐2 associated liver injury was generally mild and did not exceed >5 times the upper reference limit, therefore not fulfilling the diagnostic criteria for hypoxic hepaitis.^35^ These findings were also obtained in critically ill patients referred to the ICU, suggesting that even in cases of severe respiratory failure during SARS‐CoV‐2 infection, the adequate oxygen supply to the liver is ensured by compensatory mechanisms.^35^, ^36^, ^39^, ^149^, ^150^, ^151^, ^152^, ^153^, ^154^

## 7 DRUG‐INDUCED LIVER INJURY

At the beginning of the COVID‐19 outbreak, evidence‐based drug therapy was not available. Over the course of 8 months, multiple studies were performed allowing us to give scientifically valid recommendations for the treatment of SARS‐CoV‐2 infection. In the meantime, various antiviral (remdesivir, lopinavir/ritonavir), antibiotic (macrolids), antimalaria/antirheumatic (hydroxychloroquine), immunomodulating (corticosteroids, tocilizumab) and antipyretic (acetaminophen) drugs have been used in clinical studies or in an off‐label fashion. For most of these drugs (eg ritonavir, remdesivir) a hepatotoxic potential has already been confirmed in in vitro/in vivo experiments and in their respective registration studies. Moreover, corticosteroid therapy, which is now recommended by the WHO in patients with severe SARS‐CoV‐2 infection,^155^ is also clearly associated with steatosis or glycogenosis.^156^ Recently, the first case of DILI associated with tocilizumab use in a COVID‐19 patient has been reported.^62^ Tocilizumab undergoes minimal hepatic metabolism, and the most probable etiology for its hepatotoxic effect is the interference with the IL‐6 pathway, which plays a key role in hepatic regeneration.^157^

## 8 THE GUT‐LIVER AXIS AS THE POTENTIAL ROUTE FOR SARS‐COV‐2 HEPATIC INFECTION

Since SARS‐CoV‐2 infection affects also the gastrointestinal (GI) tract,^158^ a significant proportion of COVID‐19 patients experience gastrointestinal symptoms, including diarrhea (2%‐35.6%), nausea (1%‐17.3%) and vomiting (1%‐6.4%).^158^ Notably, both SARS‐CoV‐2 RNA and viable virions have been identified in stool samples of infected patients and post‐mortem.^24^, ^159^, ^160^, ^161^, ^162^ Hepatic and gastrointestinal manifestations appear more frequently in severe forms of COVID‐19 infections.^3^, ^4^, ^5^, ^163^, ^164^, ^165^ Interestingly, a recent study by Jin and colleagues showed that individuals with pre‐existing liver diseases are more susceptible to develop an intestinal phenotype upon SARS‐CoV‐2 infection.^163^ SARS‐CoV‐2 is potentially able to infect cells of the gastrointestinal tract, since ileal and colonic enterocytes co‐express ACE2 and TMPRSS2, the central proteins for viral attachment.^166^, ^167^, ^168^ Recently, viral nucleocapsid protein could be demonstrated within enterocytes by immunohistochemistry.^24^ The Human Protein Atlas database further corroborates these observations, with intestinal cells exhibiting the highest pattern of ACE2 expression across the whole human cell type repertoire (data available at https://www.proteinatlas.org/ENSG00000130234‐ACE2/tissue). Moreover, human intestinal organoids have been shown to be permissive to SARS‐CoV and SARS‐CoV‐2 infection.^169^ Direct gastrointestinal infection has been reported also by biopsy‐proven RNA and nucleocapsid protein detection in gastric, duodenal and rectal epithelia.^160^ Interestingly, gastrointestinal symptoms may appear before or even in the absence of manifestations in the respiratory tract.^165^ This suggests that the GI tract might be a primary site of COVID‐19 infection, and therefore that oral‐fecal transmission could be an alternative route of infection for SARS‐CoV‐2 (this has been extensively reviewed).^162^, ^170^

We would like to propose the following putative way of SARS‐CoV‐2 infection through the hepatobiliary system. COVID‐19 intestinal infection might impair the intestinal epithelial and vascular barriers, eventually leading to hepatic translocation of the virus through the portal vein. Hepatic infection might therefore start in hepatocytes, which express the required receptor binding proteins and are in direct contact with the portal circulation. Subsequently, SARS‐CoV‐2 virions exiting infected hepatocytes by transcytotic vesicular pathways could reach the bile, which has tested positive in some studies,^76^ although this remains controversial.^49^ As a result, cholangiocytes might also get in contact with and infected by SARS‐CoV‐2. Since the biliary tract provides a direct link between liver and gut, SARS‐CoV‐2 may thereby reach and infect the intestine via bile, causing in turn a second wave of infection.

Thus, the here proposed speculative mechanism could generate a vicious circle, which increases the chances of survival for the virus and might explain the worse overall outcome in patients manifesting hepatic and intestinal symptoms upon SARS‐CoV‐2 infection. On the other hand, COVID‐19 with fatal outcome seems to be associated with severe damage of lung tissue, whereas the intestines are only mildly altered, most commonly by focal ischaemic changes in the intestinal mucosa.^24^ Whether biliary tropism and requirement of bile/bile acids for viral attachment and entry into cholangiocytes and enterocytes^171^, ^172^ also play a role for SARS‐CoV‐2 remains to be determined. Given the functional and physiological similarities between bile ducts and ducts of the exocrine pancreas, and the observations concerning a potential pancreatic involvement in COVID‐19 infection,^49^, ^173^, ^174^ the research of a common mechanism allowing infection of the two tissues might help in uncovering further determinants of SARS‐CoV‐2 tropism.

## 9 CONCLUSIONS AND PERSPECTIVES

Over the last months, several studies have highlighted the potential role of liver involvement in COVID‐19 infection and pathology. In this review, we analysed the published experimental and clinical findings concerning SARS‐CoV‐2 and previous coronavirus pandemics and proposed mechanisms concerning a putative SARS‐CoV‐2 hepatic tropism and the interplay between cytopathic and systemic effects in hepatic COVID‐19 pathophysiology.

Elevated liver enzymes reflecting hepatic injury are common in COVID‐19 patients both with and without chronic liver diseases.^35^, ^38^, ^43^, ^44^ Interestingly, while early clinical studies identified significant raises exclusively in serum ALT and AST upon SARS‐CoV‐2 infection, which reflect hepatocellular damage, recent investigations and metanalyses also highlighted significant increases in ALP and γ‐GT and therefore cholangiocellular injury.^35^, ^45^ However, it is still not clear whether elevated serum liver biochemistries are causative for the worse outcome, or a consequence of the severe disease course.

In COVID‐19 patients without pre‐existing hepatic conditions who experienced liver damage, the injury is mostly mild. However, given the central role of the liver in endo‐ and xenobiotic/drug metabolism, coagulation, albumin and acute phase reactant production, hepatic dysfunction may impact on systemic disease pathophysiology of COVID‐19. Long‐term follow‐up studies are required to explore potential long‐term sequels of SARS‐CoV‐2 infection such as fibrosis.

Crucial questions remain open and need to be answered by future research: Which specific hepatic cells are infected by SARS‐CoV‐2? Which molecular processes are dysregulated by the infection? What is the real contribution of direct cytopathic effects, cytokine storm, DILI or hypoxia in hepatic dysfunction? By which means could liver injury promote respiratory failure and predispose to a severe course of COVID‐19?

The establishment of international registries collecting clinical reports of patients with liver diseases also tested positive for COVID‐19, such as the COVID‐Hep^175^ and the SECURE‐Cirrhosis,^176^ together with molecular and translational research will surely help us shed some light on these intriguing questions and to set up more effective hepatoprotective programs for future pandemics.

## CONFLICT OF INTERESTS

The Medical Universities of Graz and Vienna have filed patents for the medical use of *nor*UDCA and MT is listed as co‐inventor. MT has served as a speaker for Falk Foundation, Gilead, Intercept and MSD; he has advised for Albireo, BiomX, Boehringer Ingelheim, Falk Pharma GmbH, Genfit, Gilead, Intercept, Jannsen, MSD, Novartis, Phenex, Regulus and Shire. He further received travel grants from Abbvie, Falk, Gilead and Intercept and research grants from Albireo, CymaBay, Falk, Gilead, Intercept, MSD and Takeda. SL has received personal fees from Roche, AstraZeneca, Novartis and Biogena outside the submitted work, Authors not named here have disclosed no conflicts of interest.

## AUTHORS’ CONTRIBUTION

MT and ADN planned the project, MT, AND and MS outlined the content and contributed to writing and editing of the manuscript. ADN, MB and EDM contributed to the research, discussion of content and writing of the molecular basic science sections of the manuscript. MT, MS and SFL contributed to the research, discussion of content and writing of the clinical sections of the manuscript. ADN, MB and MS generated the figures of the manuscript. All authors corrected and approved the final version of the manuscript.
